# Dual role of CD44 isoforms in ampullary adenocarcinoma: CD44s predicts poor prognosis in early cancer and CD44ν is an indicator for recurrence in advanced cancer

**DOI:** 10.1186/s12885-015-1924-3

**Published:** 2015-11-16

**Authors:** Cheng-Lin Wu, Ying-Jui Chao, Ta-Ming Yang, Yi-Ling Chen, Kung-Chao Chang, Hui-Ping Hsu, Yan-Shen Shan, Ming-Derg Lai

**Affiliations:** 1 Department of Pathology, National Cheng Kung University Hospital, College of Medicine, National Cheng Kung University, Tainan, Taiwan; 2 Department of Surgery, National Cheng Kung University Hospital, College of Medicine, National Cheng Kung University, Tainan, Taiwan; 3 Institute of Clinical Medicine, College of Medicine, National Cheng Kung University, Tainan, Taiwan; 4 Department of Surgery, Tainan Municipal Hospital, Tainan, Taiwan; 5 Department of Senior Citizen Service Management, Chia-Nan University of Pharmacy and Science, Tainan, Taiwan; 6 Department of Biochemistry and Molecular Biology, College of Medicine, National Cheng Kung University, Tainan, Taiwan; 7 Institute of Basic Medical Sciences, College of Medicine, National Cheng Kung University, Tainan, Taiwan; 8 Center for Infectious Diseases and Signaling Research, College of Medicine, National Cheng Kung University, Tainan, Taiwan

**Keywords:** Ampullary cancer, CD44, CD44ν3-10, CD44ν6-10, Ingenuity pathway analysis, Pancreatic invasion

## Abstract

**Background:**

Although postoperative adjuvant chemoradiotherapies prevent recurrence for some patients with ampullary cancer, the recurrence rate is as high as 29 % in patients with stage I cancer. In an effort to identify predictors of recurrence in patients with ampullary adenocarcinoma, we investigated the clinical value of assessing standard and variant forms of CD44.

**Methods:**

Immunohistochemistry staining and reverse-transcription polymerase chain reaction (RT-PCR) was used to detect standard and variant forms of CD44 in samples of ampullary adenocarcinoma. The cDNA microarray analysis comparing tumors with or without pancreatic invasion was undertaken and analyzed by Ingenuity Pathway Analysis.

**Results:**

The standard CD44 (CD44s) isoform was detected in 76 of 98 patients with ampullary adenocarcinoma, and the negative or weak expression of CD44s was correlated with pancreatic invasion, lymphovascular invasion, advanced stage and bone metastasis. Moderate to dense expression of CD44s was correlated with shorter overall survival in patients with localized cancer (T1 or T2 disease, *P* = 0.0268). The patients with advanced cancer (T3 or T4 disease) and moderate or dense CD44s expression had a trend toward better survival. Alternative splicing of CD44 was confirmed using RT-PCR, which revealed that the CD44ν3-10 isoform was only expressed in patients with cancer recurrence. Fold change of CD44ν6-10 was also increased. In addition, networks containing CD44, vascular endothelial growth factor (VEGF), epidermal growth factor receptor (EGFR), transforming growth factor-β (TGF-β), matrix metalloproteinase 2 (MMP2), AKT, extracellular signal-regulated protein kinase 1 and 2 (ERK1/2), p38 MAPK, activated protein 1 (AP1)‚ and CTNNB1 were constructed after comparing microarray data from patients with and without pancreatic invasion.

**Conclusions:**

Whereas CD44s functions as tumor-promoting oncoprotein in early localized ampullary adenocarcinoma, CD44 variants are expressed in advanced cancer and patients with recurrence. Regional invasiveness and distant metastasis of ampullary cancer is controlled by a complex interacting network.

**Electronic supplementary material:**

The online version of this article (doi:10.1186/s12885-015-1924-3) contains supplementary material, which is available to authorized users.

## Background

The ampulla of Vater is located over the second portion of duodenum and around the orifice of the common bile and pancreatic ducts. In a national population-based study conducted in United States, the five-year survival rate of ampullary carcinoma was 36.8 % after resection [1]. The predictors of survival include cancer stage, differentiation, histological type, lymph node metastasis, pancreatic invasion, tumor size, lymphovascular invasion, perineural invasion and coexisting adenomas [2–4]. Although postoperative adjuvant chemoradiotherapies may prevent cancer recurrence for some patients [5], the recurrence rate is 54 % after multimodality treatment and is as high as 29 % in patients with stage I ampullary cancer [6]. Therefore, identification of predictors for recurrence in patients with early ampullary cancer is imperative.

The underlying cause of ampullary cancer is complex, involving mutation of oncogenes, silencing of tumor suppressors, alteration of cell adhesion molecules, and activation of tumor-associated macrophages [7–9]. Recently, a role for cancer stem cells (CSCs), which are multipotential cells with resistance to cytotoxic therapy, has been suggested [10]. The possible markers of CSCs in gastrointestinal cancer include CD44, epithelial cell adhesion molecule (EpCAM), CD166, CD133, CD24, and aldehyde dehydrogenase 1 [10]. Crosstalk between different oncogenic pathways, including Wnt, Notch, Hedgehog, and bone morphogenetic protein (BMP) pathways, are important for maintaining the stem cell characteristics [11]. Increased expression of CD24 is found in ampullary carcinomas than ampullary adenoma or normal mucosa of periampullary duodenum [12]. Moreover, CD44, CD133, CD166‚ and EpCAM are considered markers of CSCs in colorectal cancer [10], and their expression is increased in ampullary carcinoma as compared with ampullary adenoma or normal mucosa [13].

CD44 is a membrane receptor for hyaluronic acid and works in process of epithelial-to-mesenchymal transition (EMT) and assembly of stem cell niches in cancer [14]. Knockdown of CD44 increases cell compliance, enhances migration potential, facilitates tumor growth and promotes lung metastasis [15]. Interaction of CD44 with growth factor receptors stimulates the proliferation and invasion of cancer cells; however, its interaction with ezrin/radixin/moesin proteins activates the tumor suppressor, merlin, to inhibit cancer growth [16]. Different functions of CD44 occur as a result of alternative splicing. The standard CD44 (CD44s) isoform is present on the membranes of most vertebrate cells and alternative splicing generates several variants (CD44ν) are only expressed on some epithelial cells in normal physiological conditions [17]. The roles of CD44s and its isoforms are different in cancer [18]. For example, overexpression of CD44s or CD44ν predicts poor prognosis in colorectal cancer [19]. In contrast, CD44s suppresses metastasis but CD44ν7-10 facilitates invasion in prostate cancer [20]. Down-regulation of CD44s and CD44ν6 is associated with advanced cancer stage and poor prognosis in patients with ampullary cancer [21].

Although expression of CD44s is detected in ampullary cancer [21], function of CD44s or CD44ν remains unclear. The aim of the present study was to evaluate the expression of CD44 in patients with ampullary adenocarcinoma. We hypothesize that expression of CD44s in early ampullary adenocarcinoma promotes recurrence, and the interaction between CD44ν and other oncogenic pathways enhances metastasis in patients with advanced ampullary adenocarcinoma.

## Methods

### Study participants

Patients who were diagnosed with ampullary adenocarcinoma at the National Cheng Kung University Hospital from April 1989 to January 2008 were enrolled after obtaining a formal written informed consent. Patients with other cell types or those without a definite diagnosis were excluded. Patient demographics, histopathological findings and outcomes were recorded from retrospective chart review. All patients received follow-up and imaging studies annually. Chart review was recorded until October 2013. The overall survival rate was defined as the period from surgery until death. This study was approved by the Institutional Review Board of the National Cheng Kung University Hospital (NCKUH IRB number: ER-95-42).

### Immunohistochemistry

Serial 5 μm-thick sections were cut from formalin-fixed, paraffin-embedded samples. The sections were deparaffinized in xylene and rehydrated in graded alcohol. Endogenous peroxidase activity was blocked with 3 % hydrogen peroxide in methanol. For heat-induced epitope retrieval, the sections were immersed in 10 mM citrate buffer and heated under pressure. The sections were next incubated overnight at 4 °C with an anti-CD44s monoclonal antibody (1:750, clone 2C5, R&D, Abingdon, United Kingdom), which recognizes all forms of CD44 [17]. The sections were next incubated with the avidin-biotin complex reagent (DAKO, California, United States) and final color development was achieved with 3-amino-9-ethyl carbazole (Zymed, California, United States). The sections were counterstained with Mayer’s hematoxylin and then mounted.

The immunohistochemistry results were scored by determining the semi-quantified proportion of cancer cells with membranous staining for CD44s in five high-power fields. CD44s expression was categorized as negative (<5 % cancer cells), weak (5 %–25 %), moderate (25 %–50 %), and dense (>50 %). Each lesion was observed and scored by the same researcher (HP Hsu). Staining in non-neoplastic tissue showed dense CD44s expression in the epithelial cells of the pancreatic and bile ducts, which provided an internal control in sections with negatively staining tumors.

The histological type was defined as intestinal or pancreaticobiliary type according to columnar cells, glandular structure, and stromal components in hematoxylin and eosin stain. Based on the updated tumor classification of World Health Organization, the former consists of simple or cribriformed tubular glands similar to those of colonic adenocarcinomas, while the latter is composed of single-layered, simple or branching glands associated with an abundant desmoplastic stroma. Nuclear pseudostratification is generally absent in pancreaticobiliary type of ampullary adenocarcinoma [22]. Typical markers of intestinal type of ampullary adenocarcinoma were examined in several specimens, including cytokeratin 20 (CK20) and CDX2 (Additional file 1: Figure S1B & 1C). Expression of CK20 and CDX2 is absence in pancreaticobiliary type of ampullary cancer (Additional file 1: Figure S1E & 1F).

### Semi-quantitative reverse transcription polymerase chain reaction (RT-PCR)

Total RNA was extracted from the fresh ampullary adenocarcinoma and normal duodenal samples using an mRNA isolation system (Qiagen, Hilden, Germany). Single-stranded cDNA was synthesized from mRNA with oligo-dT as the random primer (Promega, Wisconsin, United States). The cDNA was amplified with the primers specific for β-actin, CD44s, CD44ν3, CD44ν6, CD44ν7 and CD44ν9 as previously described (Additional file 2: Table S1) [23]. PCR products were analyzed by agarose gel electrophoresis and compared with the β-actin band.

### cDNA Microarray

Five pairs of ampullary adenocarcinoma and normal duodenal tissues isolated from the same patients were sent for cDNA microarray analysis (Additional file 2: Table S2). The RNA from normal duodenal tissue was labeled with Cy3 (CyDye, PerkinElmer, Waltham, MA USA), and the RNA from ampullary adenocarcinoma was labeled with Cy5 during the *in vitro* transcription process. A total of 0.3 μg of Cy-labeled cRNA was fragmented to an average size of 50–100 nucleotides and hybridized to an Agilent SurePrint G3 Human GE 8 × 60 K microarray (Agilent, Santa Clara, CA, USA) at 65 °C for 17 h. The microarrays were scanned at 535 nm for Cy3 and 625 nm for Cy5. Scanned images were analyzed and quantified. The results were substantially normalized using the rank-consistency-filtering LOWESS method, and the data was analyzed using GeneSpring software (Agilent). The Ingenuity Pathway Analysis (IPA6.0; Ingenuity Systems, Redwood City, CA, USA; www.ingenuity.com) was used to identify networks of interacting genes.

### Statistical analysis

All statistical analyses were carried out using SPSS version 12.0 (SPSS Institute, Chicago, IL, USA). Univariate analysis was performed using the chi-square test. Continuous variables that did not follow normal distribution were compared using the nonparametric Mann–Whitney test. Associations between the immunohistochemistry staining and clinical outcomes were assessed using the Kaplan-Meier method, and significance was tested using the log-rank test. The Cox proportional hazard regression model was used to evaluate multiple predictors of overall survival. Each model included age and sex as covariates. *P*-values < 0.05 were considered statistically significant.

## Results

### CD44s expression in ampullary adenocarcinoma

**Fig. 1:**
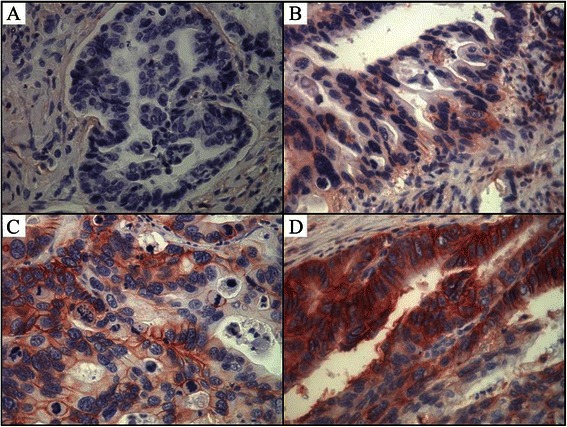
Expression of CD44s in ampullary cancer. Representative immunohistochemistry analysis of membranous CD44s staining (×400) showing (**a**) negative immunoreactivity, (**b**) weak expression of CD44s in < 25 % of ampullary cancer cells, (**c**) moderate expression of CD44s in 25 to 50 % of ampullary cancer cells, and (**d**) dense expression of CD44s in > 50 % of ampullary cancer cells

From April 1989 to January 2008, a total of 98 patients (45 females and 53 males) with ampullary adenocarcinoma were enrolled, including two patients with liver metastasis who received pancreaticoduodenectomy and metastectomy with curative intent. Dense expression of CD44s was observed in 20 patients, moderate expression in 23 patients, weak expression in 32 patients, and loss of CD44s expression in 23 patients (Fig. 1). As shown in Table 1, after grouping the patients by CD44s expression (i.e., negative or weak [<25 %] versus moderate or dense [≥ 25 %]), negative or weak expression of CD44s was significantly associated with positive lymphovascular invasion (*P* = 0.006), pancreatic invasion (*P* = 0.004), and advanced pathological tumor stage or AJCC TNM stage (*P* = 0.034 and *P* = 0.019, respectively). Alternatively, patients with moderate or dense CD44s expression had favorable disease profiles, including negative lymphovascular invasion, negative pancreatic invasion, and early tumor stage or AJCC TNM stage (Table 1). Histological type (intestinal or pancreaticobiliary type) was not correlated with CD44s expression (*P* = 0.139, Table 1).

**Table 1:** Correlation of CD44s expression with demographics and histopathological findings in patients with ampullary adenocarcinoma who underwent resection

	Extent of CD44s expression^a^		
	Negative or weak (< 25 %)	Moderate or dense (≥ 25 %)	*P*-value
Patients, number (%)	55 (56 %)	43 (44 %)	
Age, y, mean (range)	65 (32–90)	65 (35–84)	0.971
Sex			0.839
Female	26 (58 %)	19 (42 %)	
Male	29 (55 %)	24 (45 %)	
Histology type^b^			0.139
Intestinal	26 (54 %)	22 (46 %)	
Pancreaticobiliary	19 (73 %)	7 (27 %)	
Gross tumor type			0.059
Polypoid	24 (45 %)	29 (55 %)	
Ulcerative	17 (71 %)	7 (29 %)	
Mixed	14 (67 %)	7 (33 %)	
Tumor size, cm	2.1 (0.7–8.0)	2.2 (0.8–5.5)	0.625
Differentiation^b^			0.904
Well	26 (58 %)	19 (42 %)	
Moderate	24 (59 %)	17 (41 %)	
Poor	4 (50 %)	4 (50 %)	
Lymphovascular invasion^b^			0.006
Negative	13 (39 %)	20 (61 %)	
Positive	24 (75 %)	8 (25 %)	
Perineural invasion^b^			0.558
Negative	19 (56 %)	15 (44 %)	
Positive	12 (67 %)	6 (33 %)	
Resection margin			0.167
Free	48 (55 %)	40 (45 %)	
Microscopically positive	7 (78 %)	2 (22 %)	
Macroscopically positive	0	1 (100 %)	
Pancreatic invasion^b^			0.004
Negative	19 (40 %)	29 (60 %)	
Positive	34 (71 %)	14 (29 %)	
Tumor stage			0.034
pT1	3 (33 %)	6 (67 %)	
pT2	17 (44 %)	22 (56 %)	
pT3	23 (68 %)	11 (32 %)	
pT4	12 (75 %)	4 (25 %)	
Lymph node metastasis^b^			0.289
pN0	30 (52 %)	28 (48 %)	
pN1	23 (64 %)	13 (36 %)	
AJCC TNM stage			0.019
Stage I	16 (42 %)	22 (58 %)	
Stage II	27 (64 %)	15 (36 %)	
Stage III	12 (75 %)	4 (25 %)	
Stage IV	0	2 (100 %)	

Abbreviations: *AJCC TNM stage*, American Joint Committee on Cancer tumor, node, metastases staging system

^a^As determined by univariate analysis

^b^Excluding patients without detailed recorded

### CD44s expression was correlated with recurrence and survival in ampullary adenocarcinoma patients

**Fig. 2:**
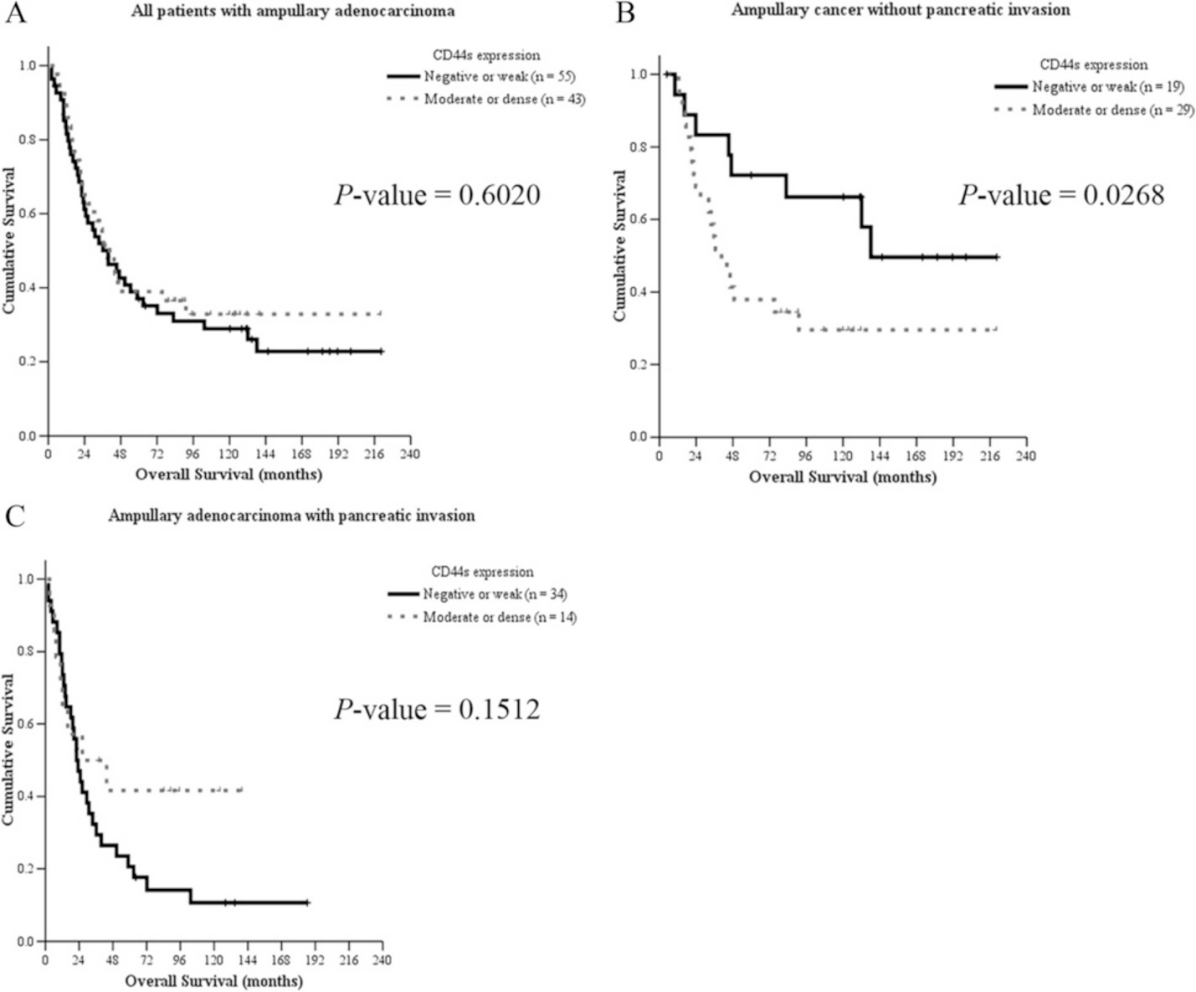
Kaplan-Meier analysis of the impact of CD44s expression on overall survival in patients with ampullary adenocarcinoma. **a** Overall survival curve of all patients with ampullary adenocarcinoma who underwent surgery by CD44s expression levels (*P* = 0.6020). **b** Overall survival curve of ampullary cancer patients without pancreatic invasion. Patients with moderate or dense CD44s expression had decreased overall survival (*P* = 0.0268). **c** Overall survival curve of ampullary cancer patients with pancreatic invasion. Patients with moderate or dense expression of CD44s had a trend toward increased survival although it was not significant (*P* = 0.1512)

In 94 patients with regular follow-up (range, 3–220 months), 60 patients developed recurrence and some patients had recurrence in two or more regions. As shown in Table 2, patients with negative or weak expression of CD44s had a higher ratio of bone metastases (*P* = 0.039). However, the 5-year overall survival rate of all patients was not associated with CD44s expression (Fig. 2a). The pancreaticobiliary type of ampullary adenocarcinoma predicts poor prognosis in some study [4]; however, the histological type was not correlated with poor survival in our series due to less patient numbers (48 patients with intestinal type and 26 patients with pancreaticobiliary type, Table 1 & Additional file 3: Figure S2A). The expression of CD44s was not associated with histological type of ampullary adenocarcinoma (Table 1), or survival in patients with intestinal or pancreaticobiliary type (Additional file 3: Figure S2B & 2C).

**Table 2:** Correlation between disease recurrence and CD44s expression in patients with ampullary adenocarcinoma who underwent radical resection

	Extent of CD44s expression		*P*-value
	Negative or weak (< 25 %)^a^ (*n* = 53)	Moderate or dense (≥ 25 %)^a^ (*n* = 41)	
Liver metastasis	16 (30 %)	9 (22 %)	0.481
Locoregional recurrence	20 (38 %)	14 (34 %)	1.000
Peritoneal carcinomatosis	7 (13 %)	4 (10 %)	0.754
Bone metastasis	9 (17 %)	1 (2 %)	0.039
Other metastasis ^b^	7 (13 %)	4 (10 %)	0.752
Total ^a,c^	35 (66 %)	25 (61 %)	0.668

^a^Excludes three patients who died due to surgical complications and one patient with stage IV disease during surgery

^b^Including brain, lung, and ovary metastases

^c^Some patients developed more than one kind of metastases

Because CD44s expression was correlated with pancreatic invasion (Table 1), which was a predictor for poor prognosis in a previous study [3], we grouped the patients according to pancreatic invasion to omit its’ interference. In patients with localized tumors without pancreatic invasion, the 5-year overall survival rate was 72.2 % in patient with negative or weak CD44s expression and 37.9 % in those with moderate or dense expression (*P* = 0.0268; Fig. 2b). In patients with pancreatic invasion, the 5-year survival rate was not correlated with the extent of CD44s expression; however, patients with moderate or dense expression of CD44s had a trend toward better survival (*P* = 0.1512; Fig. 2c).

Histological differentiation, tumor size, nodal metastases, and AJCC TNM stage were predictors of overall survival in previous studies [2–4]. Thus, a multivariate analysis based on the Cox proportional hazard model was next undertaken to identify prognostic factors that could predict overall survival in patients who had localized ampullary adenocarcinoma without pancreatic invasion (Table 3). After serial analysis, only TNM stage and CD44s expression were prognostic indicators of overall survival. Advanced TNM stage or moderate to dense CD44s expression predicted poor survival (*P* ≤ 0.001 and *P* = 0.035, respectively; Table 3).

**Table 3:** Multivariate analysis of prognostic factors for overall survival in patients with ampullary adenocarcinoma without pancreatic invasion

	Hazard ratios	95 % confidence intervals	*P*-value
Age	1.042	1.000 – 1.086	0.052
Sex			
Female	1		
Male	1.391	0.614 – 3.151	0.429
AJCC TNM Stage			< 0.001
Stage I	1		
Stage II	3.396	1.335 – 8.634	0.010
Stage III	13.483	2.449 – 74.247	0.003
Stage IV	100.752	5.462 – 1858.372	0.002
CD44s expression			
Negative or weak	1		
Moderate or dense	2.666	1.070 – 6.645	0.035

**Fig. 3:**
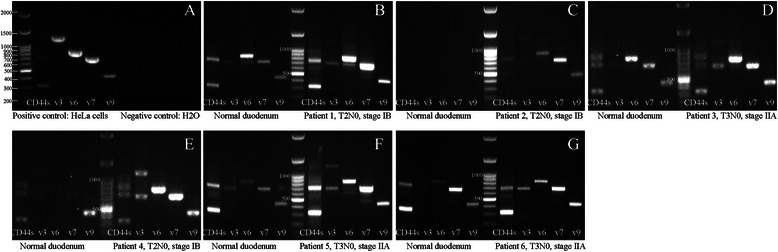
RT-PCR analysis of the expression of CD44s and its variants in ampullary adenocarcinoma. Fresh samples from six patients with ampullary adenocarcinoma and corresponding normal duodenal tissues were collected from three patients with stage IB cancer (patients 1, 2, and 4) and three patients with stage IIA cancer (patients 3, 5, and 6). Patients 1, 2, and 3 had no disease recurrence while patients 4, 5, and 6 developed recurrence. **a** HeLa cells served as a positive control. Expression of CD44s, CD44ν3, CD44ν6, CD44ν7, and CD44ν9 was compared between cancer tissue and normal duodenum. The expression patterns were different between patients without recurrence or those with recurrence

**Fig. 4:**
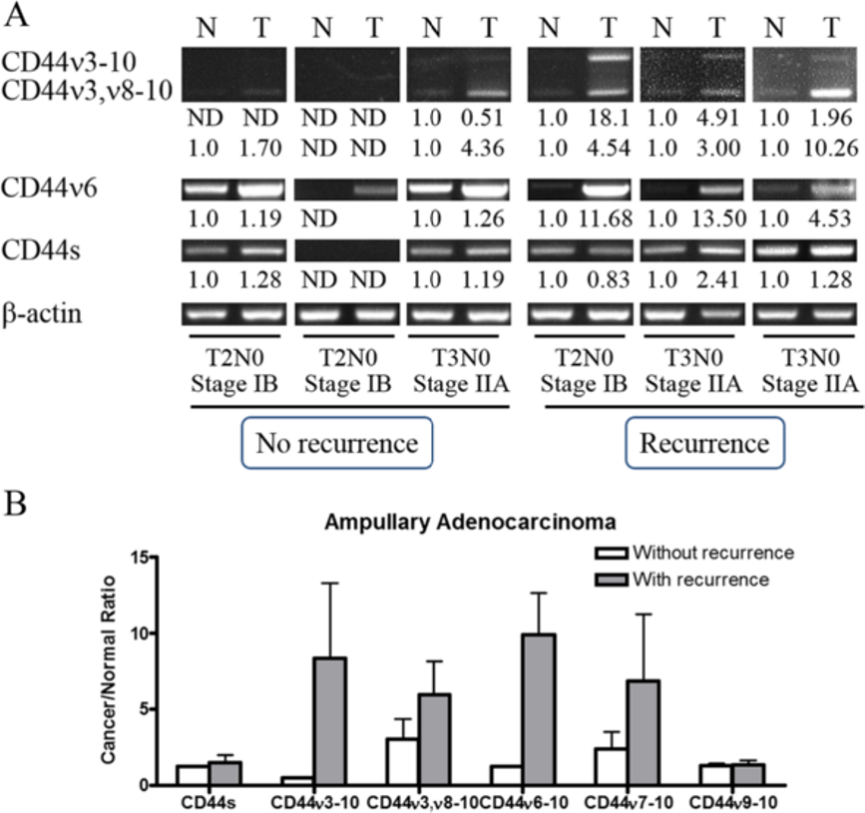
Expression of CD44s, CD44ν3-10, CD44ν6-10 and CD44ν3,ν8-10 in ampullary adenocarcinoma. **a** The expression of CD44s and its variants in ampullary adenocarcinoma and corresponding normal duodenal tissues was determined by RT-PCR. β-actin served as a positive control. The fold-change in CD44 isoform/β-actin was labeled below the band. CD44ν3-10 and CD44ν3,ν8-10 mRNA was low in patients without cancer recurrence and increased in those with cancer recurrence. Fold change of cancer/normal ratio of CD44ν6-10 was increased in those with cancer recurrence. **b** The cancer/normal ratio of CD44/β-actin expression was correlated with cancer recurrence

We next analyzed CD44s, CD44ν3, CD44ν6, CD44ν7 and CD44ν9 expression in clinical samples from six patients, including three ones with recurrence. Expression of mRNA was examined by semiquantified RT-PCR and compared between ampullary adenocarcinoma and corresponding duodenal tissues. As shown in Fig. 3, CD44ν expression was increased in cancer tissue as compared to normal duodenal tissue. In addition, the cancer/normal ratios of CD44s and CD44ν9-10 expression were similar between patients with and without recurrence (Fig. 4); however, an increased cancer/normal ratio of CD44ν3-10 and CD44ν6-10 expression was detected in the patients with recurrence (Fig. 4). These data suggested that the expression of CD44ν changed during cancer recurrence.

### Crosstalk between CD44-mediated signaling and other oncogenic pathways

In present study, the moderate or dense expression of CD44s was predictive of a better prognosis in patients with advanced cancer and pancreatic invasion (Fig. 2c). However, CD44s was not associated with cancer recurrence (Table 2), suggesting that other oncogenic pathways may be activated in these patients and cooperated with CD44ν.

Microarray analysis of five pairs of cancer and normal duodenal tissue was next undertaken. Two patients had T2N0, stage IB cancer without pancreatic invasion and one of them developed recurrence. Three patients had T3N0, stage IIA cancer with pancreatic invasion, and two had disease recurrence (Additional file 2: Table S2). After identification of genes in cancer and comparing to normal tissue, a total of 122 genes were altered in patients with recurrence but not in those without recurrence. Many of these selected genes were associated with cellular movement (Additional file 4: Figure S3).

An interaction network of genes associated with cellular movement was constructed with CD44 as the central molecule (Additional file 5: Figure S4). Analysis of proteins upstream and downstream of CD44 revealed several molecules associated with EMT or metastasis signaling in colorectal cancer, including vascular endothelial growth factor (VEGF), epidermal growth factor receptor (EGFR), transforming growth factor-β (TGF-β), matrix metalloproteinase 2 (MMP2), AKT, extracellular signal-regulated protein kinase 1 and 2 (ERK1/2), p38 MAPK, activated protein 1 (AP1) and CTNNB1 (encoded protein: β-catenin). Complex interactions among these signaling pathways may promote local invasion, distant metastasis, and recurrence of ampullary cancer rather than a single molecule, such as CD44s.

## Discussion

The five-year survival rate of patients with ampullary cancer ranges from 30 to 50 % after radical resection and adjuvant chemoradiotherapy [1–5]. Even in those with stage I ampullary cancer, 29 % of patients experience disease recurrence [6]. In the present study, negative or weak expression of CD44s was associated with positive pancreatic invasion, positive lymphovascular invasion, and bone metastasis in all patients with ampullary adenocarcinoma. Moderate to dense expression of CD44s was correlated with poor prognosis in patients who had localized cancer without pancreatic invasion (T1 or T2 disease). In addition, the cancer/normal ratio of CD44ν3-10 and CD44ν6-10 increased in the patients with cancer recurrence. Networks containing CD44 generated from cDNA microarray analysis revealed that the pathways associated with migration had a key role in pancreatic invasion and cancer recurrence of ampullary adenocarcinoma.

CD44 is a transmembrane glycoprotein, and alternative splicing generates different isoforms [18]. The roles of CD44s and its isoforms are diverse in cancer. For example, expression of CD44s and CD44ν6 enhances cell proliferation and migration and is associated with poor prognosis in colorectal cancer patients [14, 19]. CD44s is also a CSC marker and is essential in initiation of gastrointestinal cancer [10]. Increased activity of CD44 cleavage enhances mitosis and dysregulated cell cycle in gastrointestinal stromal tumor [24]. On the contrary, decreased expression of CD44s in prostate cancer and increased expression of CD44ν7-10 induced invasion, migration and independent growth of cancer cells [25]. In addition, the cytoplasmic domain of CD44 interacts with merlin to regulate actin organization, cell motility, and tumor suppression [16, 26]. Furthermore, reduced CD44ν6 and CD44s expression is correlated with poor prognosis in patients with ampullary carcinoma and Yokoyama et al. speculate that CD44 may maintain normal cell-cell adhesion to suppress metastasis [21]. Thus, the tumor-promoting ability of CD44 may be finely tuned to a particular disease condition. In the present study, moderate or dense expression of CD44s is associated with poor prognosis in patients who had ampullary adenocarcinoma without pancreatic invasion (Table 1 & Fig. 2b). In patients with advanced cancer and pancreatic invasion, moderate or dense expression of CD44s was associated with better prognosis (Fig. 2c). In addition, increased expression of CD44ν3-10 and CD44ν6-10 was only detected in all three patients with recurrent ampullary adenocarcinoma (Figs. 3 & 4). Taken together, these data suggested that the tumor-promoting ability of CD44s was activated in early ampullary adenocarcinoma. Inhibition of CD44s expression by merlin or switch to CD44ν by alternative splicing in advanced ampullary cancer may be responsible for the positive pancreatic invasion, positive lymphovascular invasion, advanced tumor or AJCC TNM stage, and bone metastasis.

Crosstalk among the Wnt, fibroblast growth factor (FGF), Notch, BMP and Hedgehog pathways is activated in stem cells and cancer initiation [11, 27]. Activations of the TGF-β and Wnt pathways as well as secretions of EGF, MMPs, and VEGF are detected in the invasive fronts of metastatic cancer cells [28]. Furthermore, TGF-β1 activates EGFR and upregulates CD44. The interaction between EGFR and CD44 promotes EMT through AKT and ERK pathways [29]. MAP kinase pathways (MEK or p38) reduce total RNA of CD44 while p38 facilitates variant splicing (CD44ν7-10) [30]. Inversed expression of CD44ν8-10 and c-Myc in gastric cancer enhances canonical Wnt signaling [31]. In the present study, variants of CD44 increased in patients with recurrent ampullary adenocarcinoma (Fig. 4). Multiple CD44-related pathways were linked to cell migration, EMT, and metastasis, including VEGF, EGFR, TGF-β, MMP2, AKT, ERK1/2, p38 MAPK, Ap1, and CTNNB1 (Additional file 4: Figure S3). TGR-β and EGFR may activate alternative splicing of CD44 through p38 MAP kinase. CD44ν may increase phosphorylation of AKT or ERK to induce cell migration (Additional file 5: Figure S4). Interactions between CD44ν and other oncologic pathways may promote recurrence in advanced cancer.

## Conclusions

The present study stratified patients with ampullary adenocarcinoma by pancreatic invasion. Poor prognosis of patients with localized cancer (T1 or T2 disease) was associated with moderate or dense CD44s expression. Thus, immunoreactivity of CD44s may be used as predictor of poor survival in patients with early cancer. In addition, the cancer/normal ratio of CD44ν3-10 and CD44ν6-10 increased in the patients with cancer recurrence. Crosstalk of multiple CD44-related pathways may be critical in ampullary cancer.

## Additional files

Additional file 1: Figure S1. Examples of histologic type of ampullary adenocarcinoma (×100). Intestinal type of ampullary adenocarcinoma was stained with hematoxylin and eosin stain (A), CK20 (B), CDX2 (C) and pancreatobiliary type was stained with hematoxylin and eosin stain (D), CK20 (E), CDX2 (F). CK20 (cytokeratin 20) and CDX2 are markers of intestinal type of ampullary adenocarcinoma and negative in pancreatobiliary type. (TIFF 14690 kb)
Additional file 2 **Table S1.**Primers used for reverse transcription polymerase chain reaction (RT-PCR). **Table S2.** Five pairs of fresh cancer tissues and corresponding normal duodenal mucosa were examined using a cDNA microarray. Here are the demographics, pathologic stage, clinical outcomes, histological type, and CD44s expression of the five patients. (DOC 43 kb)
Additional file 3: Figure S2. Kaplan-Meier analysis of the impact of CD44s expression on overall survival in patients with ampullary adenocarcinoma. (A) Overall survival curve of patients with intestinal type of ampullary adenocarcinoma who underwent surgery by CD44s expression levels (*P* = 0.4565). (B) Overall survival curve of patients with pancreaticobiliary type of ampullary adenocarcinoma by CD44s expression levels (*P* = 0.9885). Expression patterns of CD44s were not correlated with overall survival in these two subtypes of ampullary adenocarcinoma. (TIFF 1694 kb)
Additional file 4: Figure S3. The most significant 122 genes associated with pancreatic invasion were analyzed by IPA6.0. Major canonical pathways of disease bio-functions were listed and cellular movement was the first one. (TIFF 993 kb)
Additional file 5: Figure S4. The most significant 122 genes associated with pancreatic invasion were analyzed by IPA6.0. Gene network was represented as nodes and lines between two nodes. Node shapes symbolized the functional class of the gene product: inverted bell, cytokine and growth factor; hook, enzyme; trefoil, kinase; dumbbells, transcription regulator; upward scoop, transmembrane receptor; circle, complex or other. The bar graph right to the particular molecules depicted as the relative fold change of the particular gene and the bar from left to right was represented as patient 1 to 5. The log ratio of fold change in gene expression was represented as number under the particular molecules. The intensity of node colors indicated the degree of upregulation (red) or downregulation (green) in ampullary cancer than normal duodenum. Continuous and dashed lines indicated direct and indirect interactions between molecules, respectively. Bold nodes with blue rims represented genes associated with EMT or colorectal cancer metastasis signaling. (TIFF 1884 kb)

## Abbreviations

AJCC TNM stage: American Joint Committee on Cancer tumor, node, metastases staging system
AP1: Activated protein 1
CD44s: standard CD44 isoform
CD44ν: CD44 variants
CK20: Cytokeratin 20
CSC: Cancer stem cell
EGFR: Epidermal growth factor receptor
EMT: Epithelial-to-mesenchymal transition
ERK1/2: Extracellular signal-regulated protein kinase 1 and 2
IPA: Ingenuity pathway analysis
MAPK: Mitogen-activated protein kinase
MMP: Matrix metalloproteinases
RT-PCR: Reverse transcription polymerase chain reaction
TGF-β: Transforming growth factor-β
VEGF: Vascular endothelial growth factor
