# Supplementary figures and images for: Dual role of CD44 isoforms in ampullary adenocarcinoma: CD44s predicts poor prognosis in early cancer and CD44ν is an indicator for recurrence in advanced cancer
Source: BMC Cancer. 2015 Nov 16;15:903. doi: 10.1186/s12885-015-1924-3 (PMC4647323; doi:10.1186/s12885-015-1924-3)

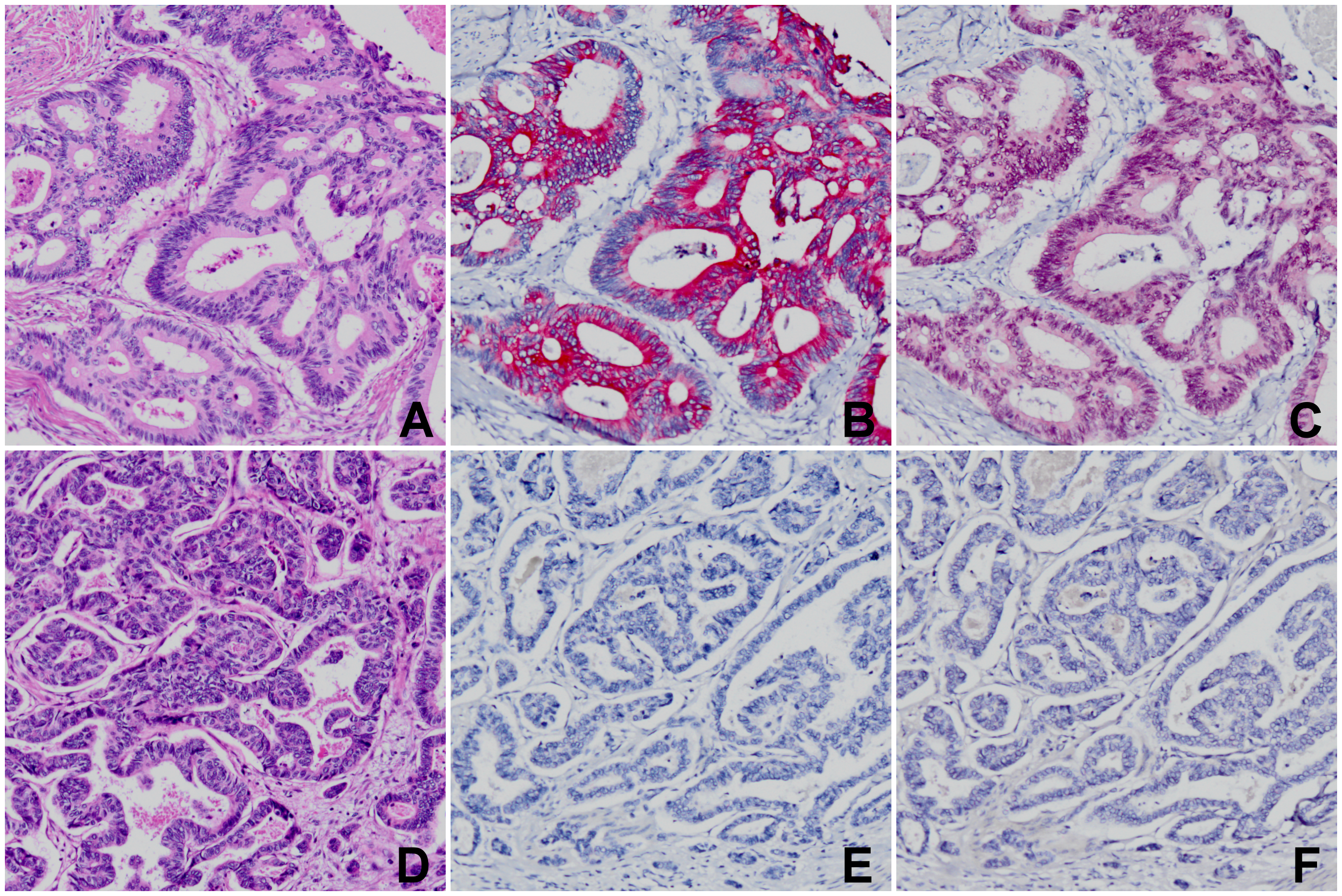

Supplement: Additional file 1: Figure S1. — Examples of histologic type of ampullary adenocarcinoma (×100). Intestinal type of ampullary adenocarcinoma was stained with hematoxylin and eosin stain (A), CK20 (B), CDX2 (C) and pancreatobiliary type was stained with hematoxylin and eosin stain (D), CK20 (E), CDX2 (F). CK20 (cytokeratin 20) and CDX2 are markers of intestinal type of ampullary adenocarcinoma and negative in pancreatobiliary type. (TIFF 14690 kb) [file 12885_2015_1924_MOESM1_ESM.tiff]

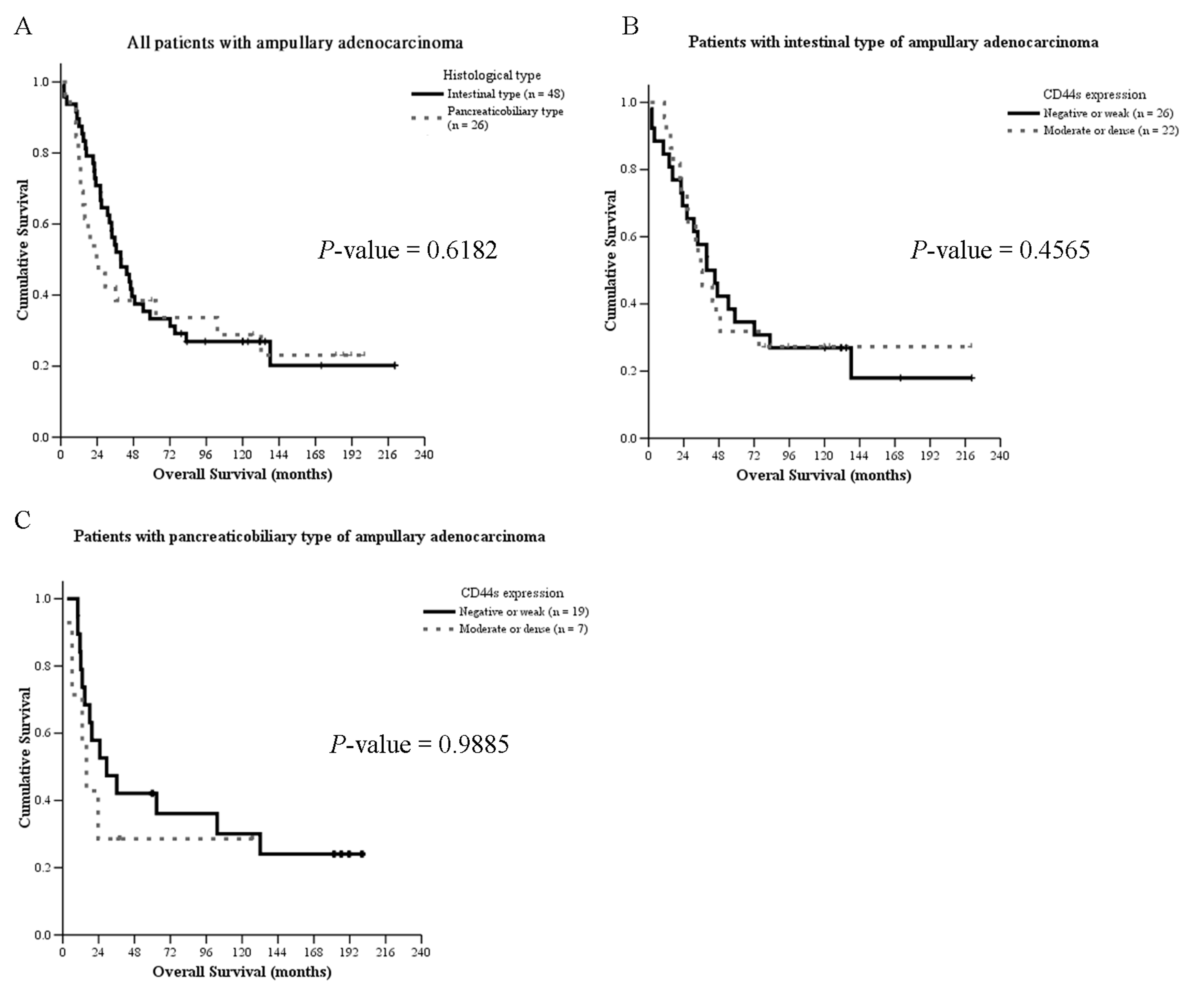

Supplement: Additional file 3: Figure S2. — Kaplan-Meier analysis of the impact of CD44s expression on overall survival in patients with ampullary adenocarcinoma. (A) Overall survival curve of patients with intestinal type of ampullary adenocarcinoma who underwent surgery by CD44s expression levels (P = 0.4565). (B) Overall survival curve of patients with pancreaticobiliary type of ampullary adenocarcinoma by CD44s expression levels (P = 0.9885). Expression patterns of CD44s were not correlated with overall survival in these two subtypes of ampullary adenocarcinoma. (TIFF 1694 kb) [file 12885_2015_1924_MOESM3_ESM.tiff]

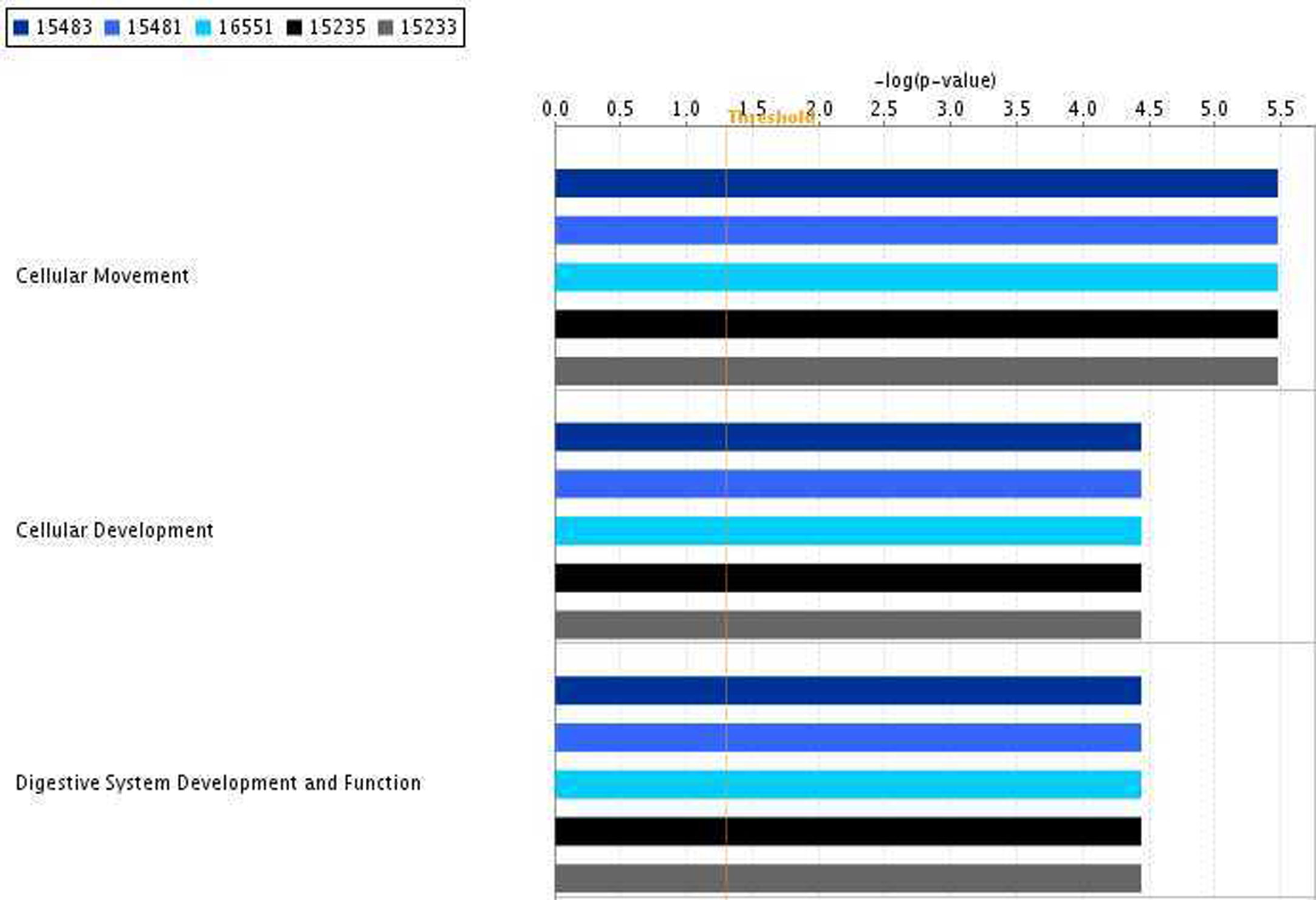

Supplement: Additional file 4: Figure S3. — The most significant 122 genes associated with pancreatic invasion were analyzed by IPA6.0. Major canonical pathways of disease bio-functions were listed and cellular movement was the first one. (TIFF 993 kb) [file 12885_2015_1924_MOESM4_ESM.tiff]

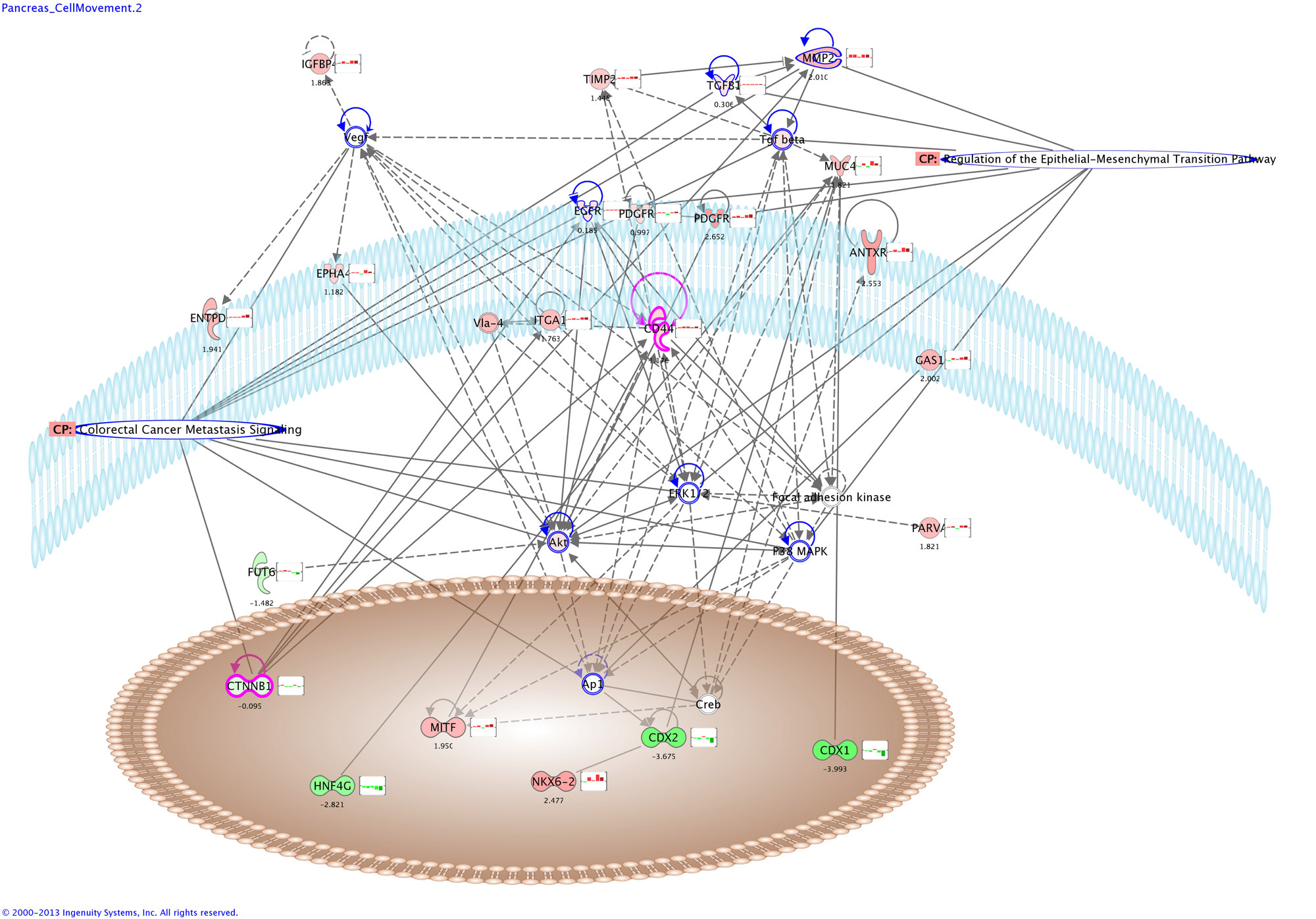

Supplement: Additional file 5: Figure S4. — The most significant 122 genes associated with pancreatic invasion were analyzed by IPA6.0. Gene network was represented as nodes and lines between two nodes. Node shapes symbolized the functional class of the gene product: inverted bell, cytokine and growth factor; hook, enzyme; trefoil, kinase; dumbbells, transcription regulator; upward scoop, transmembrane receptor; circle, complex or other. The bar graph right to the particular molecules depicted as the relative fold change of the particular gene and the bar from left to right was represented as patient 1 to 5. The log ratio of fold change in gene expression was represented as number under the particular molecules. The intensity of node colors indicated the degree of upregulation (red) or downregulation (green) in ampullary cancer than normal duodenum. Continuous and dashed lines indicated direct and indirect interactions between molecules, respectively. Bold nodes with blue rims represented genes associated with EMT or colorectal cancer metastasis signaling. (TIFF 1884 kb) [file 12885_2015_1924_MOESM5_ESM.tiff]
