# Supplementary material for: Dual role of CD44 isoforms in ampullary adenocarcinoma: CD44s predicts poor prognosis in early cancer and CD44ν is an indicator for recurrence in advanced cancer
Source: BMC Cancer. 2015 Nov 16;15:903. doi: 10.1186/s12885-015-1924-3 (PMC4647323; doi:10.1186/s12885-015-1924-3)
Supplement: Additional file 2 — Table S1.Primers used for reverse transcription polymerase chain reaction (RT-PCR). Table S2. Five pairs of fresh cancer tissues and corresponding normal duodenal mucosa were examined using a cDNA microarray. Here are the demographics, pathologic stage, clinical outcomes, histological type, and CD44s expression of the five patients. (DOC 43 kb) [file 12885_2015_1924_MOESM2_ESM.doc]

| **Supplementary Table 1.** Primers used for reverse transcription polymerase chain reaction (RT-PCR). | | |
| --- | --- | --- |
| Sense primer | | Product size |
| CD44s | AAG ACA TCT ACC CCA GCA AC | 320 bp |
| CD44ν3 | ACG TCT TCA AAT ACC ATC TC | 1166 bp (CD44ν3-10)  675 bp (CD44ν3-8-9-10) |
| CD44ν6 | CAG GCA ACT CCT AGT AGT AC | 821 bp (CD44ν6-10) |
| CD44ν7 | CAG CCT CAG CTC ATA CCA GC | 691 bp (CD44ν7-10) |
| CD44ν9 | CAG AGC TTC TCT ACA TCA CA | 432 bp (CD44ν9-10) |
| β-actin | AGC GGG AAA TCG TGC GTG | 309 bp |
| Antisense primer | |  |
| CD44s | CCA AGA TGA TCA GCC ATT CTG G |  |
| β-actin | CAG GGT ACA TGG TGG TGG TGC C |  |

| **Supplementary Table 2.** Five pairs of fresh cancer tissues and corresponding normal duodenal mucosa were examined in a cDNA microarray. Here are the demographics, pathologic stage, clinical outcomes, histological type, and CD44s expression of the five patients. | | | | | |
| --- | --- | --- | --- | --- | --- |
| No. | Age range | Stage | Clinical outcomes | Histological type | Expression of CD44 |
| 1 | 50-59 | T2N0, stage IB | No recurrence (post-OP 137 months) | Intestinal type | Moderate |
| 2 | 50-59 | T2N0, stage IB | Recurrence at post-OP 14 months, expired at post-OP 17 months | Intestinal type | Dense |
| 3 | 60-69 | T3N0, stage IIA | No recurrence (post-OP 108 months) | Intestinal type | Dense |
| 4 | 60-69 | T3N0, stage IIA | Recurrence at post-OP 6 months, expired at post-OP 17 months | Intestinal type | Not done |
| 5 | 60-69 | T3N0, stage IIA | Recurrence at post-OP 6 months, expired at post-OP 22 months | Intestinal type | Negative |
|  | | | | | |

**Figure legends of supplementary figures:**

**Supplementary Figure 1.** Examples of histologic type of ampullary adenocarcinoma (×100). Intestinal type of ampullary adenocarcinoma was stained with hematoxylin and eosin stain (A), CK20 (B), CDX2 (C) and pancreatobiliary type was stained with hematoxylin and eosin stain (D), CK20 (E), CDX2 (F). CK20 (cytokeratin 20) and CDX2 are markers of intestinal type of ampullary adenocarcinoma and negative in pancreatobiliary type.

**Supplementary Figure 2**. Kaplan-Meier analysis of the impact of CD44s expression on overall survival in patients with ampullary adenocarcinoma. (A) Overall survival curve of patients with intestinal type of ampullary adenocarcinoma who underwent surgery by CD44s expression levels (*P* = 0.4565). (B) Overall survival curve of patients with pancreaticobiliary type of ampullary adenocarcinoma by CD44s expression levels (*P* = 0.9885). Expression patterns of CD44s was not correlated with overall survival in these two subtypes of ampullary adenocarcinoma.

**Supplementary Figure 3.** The most significant 122 genes associated with pancreatic invasion were analyzed by IPA6.0. (A) Major canonical pathways of disease bio-functions were listed and cellular movement was the first one. (B) Gene network was represented as nodes and lines between two nodes. Node shapes symbolized the functional class of the gene product: inverted bell, cytokine and growth factor; hook, enzyme; trefoil, kinase; dumbbells, transcription regulator; upward scoop, transmembrane receptor; circle, complex or other. The bar graph right to the particular molecules depicted as the relative fold change of the particular gene and the bar from left to right was represented as patient 1 to 5. The log ratio of fold change in gene expression was represented as number under the particular molecules. The intensity of node colors indicated the degree of upregulation (red) or downregulation (green) in ampullary cancer than normal duodenum. Continuous and dashed lines indicated direct and indirect interactions between molecules, respectively. Bold nodes with blue rims represented genes associated with EMT or colorectal cancer metastasis signaling.
